# Feeding butter with elevated content of trans-10, cis-12 conjugated linoleic acid to lean rats does not impair glucose tolerance or muscle insulin response

**DOI:** 10.1186/1476-511X-13-101

**Published:** 2014-06-23

**Authors:** Amanda Stefanson, Loren E Hopkins, Ousama AlZahal, Ian R Ritchie, Tara MacDonald, David C Wright, Brian W McBride, David J Dyck

**Affiliations:** 1 Department of Human Health and Nutritional Sciences, University of Guelph, Guelph, Ontario N1G2W1, Canada; 2 Department of Animal and Poultry Science, University of Guelph, Guelph, Ontario N1G2W1, Canada

**Keywords:** Conjugated linoleic acid, Butter, Rats, Glucose tolerance, Insulin tolerance, Insulin-stimulated glucose uptake

## Abstract

**Background:**

Numerous studies have investigated the effects of isolated CLA supplementation on glucose homeostasis in humans and rodents. However, both the amount and relative abundance of CLA isomers in supplemental form are not representative of what is consumed from natural sources. No study to date has examined the effects of altered CLA isomer content within a natural food source. Our goal was to increase the content of the insulin desensitizing CLA_t10,c12_ isomer relative to the CLA_c9,t11_ isomer in cow’s milk by inducing subacute rumenal acidosis (SARA), and subsequently investigate the effects of this milk fat on parameters related to glucose and insulin tolerance in rats.

**Methods:**

We fed female rats (~2.5 to 3 months of age) CLA _t10,c12_ –enriched (SARA) butter or non-SARA butter based diets for 4 weeks in either low (10% of kcal from fat; 0.18% total CLA by weight) or high (60% of kcal from fat; 0.55% total CLA by weight) amounts. In an effort to extend these findings, we then fed rats high (60% kcal) amounts of SARA or non-SARA butter for a longer duration (8 weeks) and assessed changes in whole body glucose, insulin and pyruvate tolerance in comparison to low fat and 60% lard conditions.

**Results:**

There was a main effect for increased fasting blood glucose and insulin in SARA vs. non-SARA butter groups after 4 weeks of feeding (p < 0.05). However, blood glucose and insulin concentration, and maximal insulin-stimulated glucose uptake in skeletal muscle were similar in all groups. Following 8 weeks of feeding, insulin tolerance was impaired by the SARA butter, but not glucose or pyruvate tolerance. The non-SARA butter did not impair tolerance to glucose, insulin or pyruvate.

**Conclusions:**

This study suggests that increasing the consumption of a naturally enriched CLA_t10,c12_ source, at least in rats, has minimal impact on whole body glucose tolerance or muscle specific insulin response.

## Background

The most abundant conjugated linoleic acid (CLA) isomers are CLA_c9,t11_ and CLA_t10,c12_ which make up approximately 76% and 1% of total naturally occurring CLA, respectively [1,2]. CLA supplements (equal mix of CLA_t10,c12_ and CLA_c9,t11_ isomers) have become popular due to their purported ability to cause desirable changed in body mass and composition in humans [3-6]. However, it is in mice that the most dramatic loss of body fat i.e. lipoatrophy is observed in response to CLA [7-9]. Interestingly, insulin resistance has also been observed as an outcome of mixed CLA supplementation in both mice [7,8] and humans [3-6] and appears linked to the CLA_t10,c12_ isomer. Human consumption of CLA from food sources is approximately 200 mg per day, which is much lower than amounts taken through supplemental form, and orders of magnitude lower than that used in the majority of rodent studies when adjusted to body mass [10]. Whether altered CLA content in natural food sources, particularly that of the potentially detrimental CLA_t10,c12_ isomer, poses a risk in terms of developing glucose intolerance or insulin resistance is completely unknown.

No study to date has examined the effects of altered CLA isomer content derived from a natural food source, such as milk fat. CLA cannot be produced endogenously, and so the only source of CLA for humans is dietary consumption of the meat, milk and derived products of ruminant animals [11]. Dairy products are a major contributor to the dietary intake of CLA. In order to increase milk production, Canadian dairy cows are often fed a high grain, low forage ration, that may produce a condition known as subacute rumenal acidosis (SARA), which is defined as a decrease in rumenal pH below 5.6 for extended periods (4–6 hrs) during a 24-hr period [12,13]. This decrease in pH results in a significant increase in the production of the CLA_t10,c12_ isomer due to incomplete biohydrogenation of polyunsaturated fatty acids [14]. In light of the evidence that high CLA_t10,c12_ intake can potentially cause insulin resistance, we endeavored to determine whether there is a health risk related to changes in glucose tolerance and insulin response as a result of increased CLA_t10,c12_ content in dairy products.

Thus, our goal was to nutritionally manipulate the ratio of the two main CLA isomers in milk fat from dairy cows by inducing SARA, and subsequently investigate the effects of an increased content of CLA _t10,c12_ in this milk fat on parameters related to glucose and insulin tolerance in rats. To this end, rodent diets were produced containing low (10% kcal) or high (60% kcal) amounts of fat derived from SARA and non-SARA butter. Female Sprague–Dawley rats were fed CLA _t10,c12_ –enriched (SARA) butter, and non-SARA (control) butter based diets for 4 to 8 weeks. The effects of the SARA and non-SARA butter based diets on fasting blood glucose and insulin concentrations, whole body glucose and insulin tolerance, as well as insulin stimulated glucose uptake in isolated skeletal muscle were evaluated in two separate experiments after 4 to 8 weeks of feeding. We hypothesized that rats fed the high CLA _t10,c12_ (SARA) butter, but not the control (non-SARA) butter based diets, would demonstrate elevated fasting blood glucose and insulin, impaired insulin stimulated glucose uptake in skeletal muscle, as well as impaired whole body glucose and insulin tolerance. If this hypothesis is correct, subsequent studies may be warranted to determine whether consumption of dairy products that are enriched in CLA _t10,c12_ increases the risk of impaired glucose and insulin tolerance in humans.

## Methods

### Ethics statement

All procedures were carried out in accordance with the recommendations of the Canadian Council of Animal Care, and were approved by the Animal Care Committee at the University of Guelph (Animal Utilization Protocols 09R046 and 1515). All surgeries on rodents were performed under sodium pentobarbital anesthesia and all efforts made to prevent discomfort and suffering.

### Dairy cows and rations

Six lactating Holstein cows housed at the Ponsonby Dairy Research Centre, University of Guelph, Guelph, Ontario were used in this study. The cows were randomly assigned to one of two dietary treatments for a total of 21 days: a high-forage diet (n = 4 cows) or a SARA-inducing high-grain diet (n = 2 cows). The high-forage diet was designed to promote a stable rumen environment and maintain an optimal rumenal pH. This was achieved by feeding a total mixed ration with high effective fiber and low grain levels (10% of DM (dry matter) high moisture corn). In contrast to this, the SARA diet provided a lower but adequate amount of fiber, and also included a combination of soybean oil (2% of DM) and readily fermentable carbohydrates in the form of wheat and barley pellets (20% of DM). The oil and grain pellets were mixed and fed as a top-dressing. This diet was intended to create a condition in which cows would experience SARA-related alterations in rumen microbial fermentation and a subsequent shift in the biohydrogenation pathway, leading to elevated levels of CLA _t10,c12_ in milk fat. Cows were fed twice daily, and received the SARA-inducing top dressing in the morning feed between 800–900 h. All animals were handled and cared for in accordance with the Canadian Council on Animal Care regulations, and as approved the Animal Care Committee at the University of Guelph.

### Milk collection and processing

Milking of all cows took place twice daily at 0500 and 1600 h. Milk collection for the experiment took place on days 13 through 21 and was performed on 7 separate days throughout this period. Each cow was milked with separate bucket milkers to avoid cross-contamination between treatments. The milk from each animal was then poured into stainless steel canisters, immediately placed on ice, and transferred to the University of Guelph Food Science Department where it was pooled by treatment and processed to make unsalted butter. The fatty acid profile of the butter was analyzed as previously reported [15].

### Study design

This study was conducted to address two objectives. The first objective was to determine whether feeding rats diets containing high and low amounts of control (non-SARA) and SARA butter for a relatively short period of time (4 weeks) was sufficient to alter basic indices of insulin sensitivity (fasting blood glucose and insulin), as well as insulin response and glucose uptake in skeletal muscle, which is by mass the largest tissue responsible for the clearance and management of blood glucose. Our initial findings indicated little effect of the SARA-based butter on our measured parameters. We therefore conducted further experiments to address our second (follow-up) objective, which was whether a more prolonged exposure to a high-SARA butter diet would lead to impaired glucose tolerance and insulin resistance. To this end, we fed rats diets high in control (non-SARA) and SARA butter (60% kcal), for an extended period (8 weeks) on whole body indices of glucose tolerance and insulin sensitivity, including glucose, insulin and pyruvate tolerance tests. The effects of these butter-based diets were compared to low fat (10% kcal) control and high (60% kcal) lard based diets, which have predictable effects on glucose and insulin tolerance. In our experience, 4–8 weeks feeding of a high fat (lard) based diet is sufficient to induce skeletal muscle insulin resistance [16,17] in lean female rats. Due to limited quantities of our custom manufactured butter, we were able to utilize 14–15 rats per dietary group to examine our initial objective, but only 5 rats per group to examine our second objective.

We chose to use lean rats, rather than mice, for this study as mice demonstrate a rather unique lipodystrophic response to CLA supplementation [7-9] which does not occur in humans. The majority of studies examining the effects of CLA on insulin sensitivity and glucose tolerance in rats have utilized the fa/fa Zucker rat. While we recognize the value of the Zucker rat as a model for obesity and diabetes, we felt that as an initial study examining the effects of naturally modified CLA content on glucose tolerance and insulin sensitivity, a more “modest” model would be more suitable. Our subsequent studies will examine the outcome of feeding butter with altered CLA profiles on fa/fa Zucker rats.

### Objective 1 - Shorter term effects of SARA vs. non-SARA butter on fasting glucose and insulin, and muscle insulin response

#### Rodents and diets

Female Sprague–Dawley rats (approximately 120 g; Charles River Laboratories, Quebec, Canada) were group-housed in a controlled environment with a reverse 12:12-h light–dark cycle during a 5 day acclimation period. During this time rats had ad libitum access to Purina standard rodent chow and fresh water. Rats were then placed in individual cages and randomly assigned to one of 4 dietary treatments (n = 14-15 per group): low fat control (non-SARA) butter diet (10% kcal from control butter), high fat control butter (60% kcal from non-SARA butter), low fat SARA butter diet (10% kcal from SARA butter), or high fat SARA butter diet (60% kcal from SARA butter). Rats remained on these diets for 4 weeks. The diets were made by mixing the control and SARA butter with a powdered diet premix (D12492px) formulated by Research Diets Inc. (New Brunswick, NJ, USA). Fresh batches of each diet were mixed every few days throughout the trial. Rodents were fed fresh food each day in the morning and weighed twice weekly. In this first study, animals were pair-fed across all four dietary treatments in order to minimize potential differences in weight gain. Following 4 weeks of feeding, rats were fasted overnight before experimental procedures were performed. All procedures were approved by the Animal Care Committee at the University of Guelph.

#### Tissue and blood sampling

##### Muscle isolation

Rats were anesthetized with an intraperitoneal injection of pentobarbital sodium (5 mg/100 g body mass) and the hindlimb soleus (SOL) muscle was carefully divided into longitudinal strips using a 27-gauge needle. The SOL strips were then incubated to measure glucose transport under basal and maximal insulin stimulated conditions as we have previously done [16,17].

##### Glucose transport

Upon excision, SOL strips were immediately placed in pre-gassed vials (95% O_2_, 5% CO_2_) in a gentle shaking bath (30°C) and equilibrated for 30 minutes in KHB (0.1% fatty acid free BSA) containing 8 mM glucose and 32 mM mannitol, in the absence or presence of insulin (10 mU/mL). Strips were then washed twice (10 min each) with glucose-free KHB (4 mM pyruvate, 36 mM mannitol). After washing, SOL strips were incubated for 20 min (insulin-stimulated, 10 mU/mL) or 40 min (basal) in KHB (4 mM pyruvate, 8 mM 3-O-[^3^H] methyl-D-glucose (800 μCi/mmol), 28 mM [^14^C]mannitol (60 μCi/mmol)]. Muscles were blotted, trimmed of tendons, weighed, and finally digested at 95°C for 10 min in 1 mL NaOH (1 M). 200 μL aliquots of muscle digest were placed in 7 mL plastic scintillation vials in duplicate, 5 mL of CytoScint scintillation cocktail was added to each vial, and samples were left to quench overnight. Intracellular 3-O-[^3^H] methyl-D-glucose was quantified and used to calculate glucose transport as described previously (23).

##### Insulin signaling proteins

Sol strips were first equilibrated in a similar manner to the first step of the glucose transport assay (above), but without insulin. Strips were then incubated for 10 min in the presence or absence of insulin (10 mU/mL) for the determination of phosphorylated (activated) Akt. Once incubation was complete, the Sol strips were immediately frozen and stored in liquid N_2_ until future Western blot analyses.

##### Blood

Terminal blood collection after an overnight fast was performed following skeletal muscle extraction via cardiac puncture. A glucometer reading (OneTouch Ultra 2, LifeScan Inc., Milpitas, CA, USA) of whole blood glucose was made at this time. Blood samples were collected in heparinized tubes and centrifuged at 9 300 g for 5 minutes at 4°C. Plasma was stored at −80°C for analysis of glucose (via glucometer) and insulin (rat insulin RIA kit; Millipore, St. Charles, MO, USA).

#### Western blot analyses

Sol muscle (~40 mg) was homogenized (1:9 w/v dilution) in ice-cold buffer suitable for protein extraction and preserving phosphorylation states of proteins, containing 50 mM Tris (pH 7.5), 1 mM EDTA, 1 mM EGTA, 50 mM NaF, 5 mM sodium pyrophosphate, 10% (vol/vol) glycerol, 1% (vol/vol) Triton X-100, 2 mg/ml leupeptin, 2 mg/ml aprotinin, 2 mg/ml pepstatin, 1 mM dithiothreitol, and 1 mM phenylmethylsulfonyl fluoride. Homogenates were sonicated for 5 seconds and centrifuged at 20,000 *g* for 20 min at 4°C. The supernatant was removed, and protein content was determined using BSA as standards. Fifty micrograms of this whole tissue lysate protein were solubilized in 4X Laemmeli’s buffer and boiled at 95°C for 5 min, resolved by SDS-PAGE, and wet transferred to polyvinylidene difluoride (PVDF) membranes (1 h, 100 V). The membranes were blocked for 1 h in 7.5% BSA and then incubated for 1 h with a primary antibody for Ser^473^ (Santa Cruz Biotechnology, Santa Cruz, CA) phosphorylated Akt. Membranes were washed and then incubated for 1 h with the secondary antibody (anti-rabbit). Membranes were washed again, and proteins were detected using enhanced chemiluminescence method. Equal loading was confirmed using nonspecific protein staining with Ponceau-S stain (Sigma Aldrich, Oakville, ON, Canada).

### Objective 2 - Longer term effects of SARA vs. non-SARA butter on whole body glucose, insulin and pyruvate tolerance tests

#### Rodents and diets

Twenty female Sprague Dawley rats (mean body weight 138 g ± 1.5 g) were obtained from Charles River (Quebec, Canada) and individually housed in a 12-hour reversed light/dark cycle with free access to water and a standard chow diet for a 1 week acclimation period. Rats were randomly assigned to one of 4 dietary treatment groups (n = 5 per group): i) low fat diet (LFD; 10% kcal from fat), ii) control (non-SARA) butter (60% kcal from fat), iii) SARA butter (60% kcal from fat), or iv) LARD (60% kcal from fat). Composition of the low fat, butter-based, and lard diets is shown in Table 1. The 60% LARD group was included as a positive control condition i.e. one in which we expected to see the development of glucose and/or insulin intolerance. In order to provide a more realistic opportunity for the various butter diets to cause any potential negative effects on glucose and insulin tolerance, rats received food ad libitum for an 8 week duration. Body mass and food intake were monitored weekly for the entire experimental period. All protocols were approved by the Animal Care Committee, and were in compliance with the guidelines outlined by the Canadian Council on Animal Care at the University of Guelph.

**Table 1:** Composition of low-fat, butter-based and LARD diets

	**Low-fat diet**		**Butter diets**		**Lard diet**	
	**g**	**kcal**	**g**	**kcal**	**g**	**kcal**
**Casein**	200	800	200	800	200	800
**L-Cysteine**	3	12	3	12	3	12
**Cornstarch**	315	1260	0	0	0	0
**Maltodextrin**	35	140	125	500	125	500
**Sucrose**	350	1400	68.8	275.2	68.8	275.2
**Cellulose**	50	0	50	0	50	0
**Soybean Oil**	25	225	25	225	25	225
**Lard**	20	180	0	0	245	2205
**Butter**	0	0	295	2165	0	0
**Mineral Mix**	10	0	10	0	10	0
**Dicalcium phosphate**	13	0	13	0	13	0
**Calcium phosphate**	5.5	0	5.5	0	5.5	0
**Potassium citrate**	16.5	0	16.5	0	16.5	0
**Vitamin mix**	10	40	10	40	10	40
**Choline bitartate**	2	0	2	0	2	0
**Dye**	0.05	0	0	0	0.05	0
**Total**	**1055**	**4057**	**824**	**4017**	**774**	**4057**
**kcal/g**		**3.85**		**4.88**		**5.24**
**%kcal from fat**		**10**		**60**		**60**

A negative control low fat diet (LFD) containing 10% kcal from fat (5.5% soybean oil and 4.5% lard; D12450B) and a positive control high fat diet (LARD) containing 60% kcal from fat (5.5% soybean oil and 54.5% lard; D12492) were obtained from Research Diets, New Jersey, USA. A premix excluding lard, but otherwise identical in composition to the LARD diet (D12492px), was used to blend custom butter-based diets with 60% kcal from fat (5.5% soybean oil and 54.5% butter). Due to the higher moisture content of butter, the amount of butter added to make up 60% kcal from fat was based on the analysis of custom butters (fat content 83.2% ± 0.45). Butter diets were prepared by blending 295 g of butter with 528.8 g of diet premix in a commercial grade food mixer (Hobart Canada, Ontario, Canada). Fresh batches of each diet were mixed every week throughout the trial and stored at 4C.

#### Tolerance tests

During the 8th (final) week, whole body glucose, insulin and pyruvate tolerance tests were performed. Tests were separated by 1–2 days.

##### Glucose tolerance test (GTT)

Rats were fasted for 6 hours and a basal blood glucose concentration was determined with blood from the tail tip using a glucometer (OneTouch Ultra 2, LifeScan Inc., Milpitas, CA, USA). A glucose bolus (2 g glucose/kg body weight) was administered by intraperitoneal injection and blood glucose concentrations were evaluated at 15, 30, 45, 60, 90 and 120 minutes post injection.

##### Insulin tolerance test (ITT)

Basal blood glucose was determined in fed rats with blood from the tail tip as described above. Blood glucose levels were determined at 10, 20, 30, 45, 60, 90 and 120 minutes after an intraperitoneal insulin injection (0.75 U insulin/kg body weight).

##### Pyruvate tolerance test (PTT)

Rats were fasted for 6 hours and basal blood glucose was determined as described above. A pyruvate bolus (2 g pyruvate/kg body weight, adjusted to pH 7.35) was administered by intraperitoneal injection and blood glucose levels were evaluated at 15, 30, 45, 60, 90 and 120 minutes post injection.

## Statistical analyses

Statistical analyses used in this study compared means with a two-way ANOVA in the experiments examining the first objective (butter type X level of fat), and a one-way ANOVA in the experiments examining the second objective. For whole body tolerance tests, group means were compared using the area under the curve (AUC) taken from baseline blood glucose (mmol/L) for each animal; the ITT used the AUC below baseline blood glucose. A Fisher’s post-hoc test was used to compare treatment groups. All statistical analyses were calculated using Prism 5.0 software (GraphPad Software Inc. 2008, San Diego, USA). Statistical significance was accepted at p ≤ 0.05.

## Results

### Altered FA profile of milk fat (butter)

The SFA, PUFA and MUFA composition of non-SARA and SARA butters is shown in Table 2. The t10,c12 CLA isomer was increased by ~ 10-fold (p < 0.001) in the SARA butter (0.10 g/100 g FA) compared to control butter (0.01 g/100 g FA; Table 3). In fact, all measured CLA isomers demonstrated some increase in the SARA butter relative to the control butter, including the c9 t11 isomer (>2-fold), which is the most naturally abundant CLA isomer.

**Table 2:** Content of saturated, monounsaturated, and polyunsaturated fatty acids in non-SARA (control) and SARA diets (g/100 g)

	**Non-SARA Diet**		**SARA Diet**	
	**g/100 g butter fat**	g/100 g diet*	**g/100 g butter fat**	g/100 g diet*
**SFA**	71 ± 0.5	21 ± 0.2	53 ± 1.3^a^	15 ± 0.4^a^
**PUFA**	3 ± 0.1	1 ± 0.01	6 ± 0.4^a^	2 ± 0.1^a^
**MUFA**	26 ± 0.1	7 ± 0.2	41 ± 0.9^a^	12 ± 0.3^a^
**Total**	100	29	100.0	29
**n3 FA**	0.6 ± 0.01	0.2 ± 0.003	0.7 ± 0.03	0.2 + 0.01
**n6 FA**	2.0 ± 0.05	0.6 ± 0.01	2.8 ± 0.2^a^	0.8 + 0.06^a^

Data presented as mean plus/minus standard error, in g/100 g of butter fat or prepared diet (*60% kcal from butter). ^a^significantly different from non-SARA butter (p < 0.05). n = 4 batches of non-SARA butter, and 4 batches of SARA butter. SFA, saturated fatty acids; PUFA, polyunsaturated fatty acids; MUFA, monounsaturated fatty acids.

**Table 3:** Content of linoleic acid (18:2 9c, 12c) and conjugated linoleic acid isomers in non-SARA (control) and SARA diets (g/100 g)

**Fatty acid**	**Non-SARA diet**		**SARA diet**	
	**g/100 g of butter fat**	g/100 g diet*	**g/100 g of butter fat**	g/100 g diet*
18:2 9c, 12c	1.72 ± 0.03	0.51 ± 0.01	2.50 ± 0.225^a^	0.75 ± 0.06^a^
18:2 11 t, 15c	0.07 ± 0.005	0.02 ± 0.0002	0.39 ± 0.002^a^	0.11 ± 0.001^a^
**18:2 9c, 11 t**	**0.49 ± 0.15**	**0.15** ± **0.005**	**1.16 ± 0.144**^**a**^	**0.34 ± 0.04**^**a**^
18:2 9 t, 11c	0.01 ± 0.00	0.0003 ± 0.000	0.08 ± 0.004^a^	0.03 ± 0.001^a^
**18:2 10 t, 12c**	**0.01 ± 0.001**	**0.0003 ± 0.000**	**0.10 ± 0.003**^**a**^	**0.03 ± 0.001**^**a**^
18:2 9 t, 11 t and 10 t, 12 t	0.03 ± 0.004	0.01 ± 0.001	0.08 ± 0.004^a^	0.03 ± 0.001^a^
18:2 11 t, 13 t	0.01 ± 0.002	0.0003 ± 0.001	0.03 ± 0.004^a^	0.01 ± 0.001^a^
Total CLA	0.62 ± 0.05	0.18 ± 0.007	1.84 ± 0.16^a^	0.55 ± 0.04^a^

Data presented as mean plus/minus standard deviation, in g/100 g of butter fat or prepared diet (*60% kcal from butter). The main CLA isomers, 9c, 11 t and 10 t, 12c are bolded. ^a^significantly different from non-SARA butter (p < 0.05). n = 4 batches of non-SARA butter, and 4 batches of SARA butter.

### Shorter term effects of SARA vs. non-SARA butter on fasting glucose and insulin, and muscle insulin response

#### Rodent feed intake and body mass

There was no significant difference in body mass (start, ~175 g, terminal, ~270 g) or caloric intake throughout the study (week 1, ~70 kcal/day; week 4, ~85 kcal/day) amongst the groups.

#### Blood and plasma measurements

Blood and plasma measurements are shown in Table 4. There was a significant main effect for treatment (SARA vs. non-SARA) on blood glucose and plasma insulin (p < 0.05). That is, blood glucose and plasma insulin were greater in the SARA vs. non-SARA conditions, regardless of the amount of fat/butter in the diet. However, there was no significant difference in fasting blood glucose between the 4 dietary groups following the 4 week trial. Fasting plasma insulin was significantly greater in the low SARA butter fed animals compared to all other groups (p < 0.05).

**Table 4:** Blood glucose and plasma insulin concentrations after 4 weeks feeding of butter based diets

**Dietary group**	**Blood glucose (mM)**	**Plasma insulin (pM)**
Low control butter diet	9.1 ± 0.2 (13)	6.1 ± 0.3 (13)
Hhigh control butter diet	9.1 ± 0.3 (15)	5.4 ± 0.4 (15)
Low SARA butter diet	9.7 ± 0.3 (15)	7.7 ± 0.4 (15)^b^
High SARA butter diet	9.4 ± 0.4 (14)	5.2 ± 0.5 (14)
Pooled control butter diet	9.1 ± 0.2 (28)	5.8 ± 0.2 (28)
Pooled SARA butter diet	9.6 ± 0.2 (29)^a^	6.5 ± 0.4 (29)^a^

Data are presented as mean plus/minus standard error. Sample size is shown in parentheses. ^a^significantly different from pooled control butter diet (p < 0.05); ^b^significantly different from all other groups (p < 0.05).

#### Glucose transport in ex vivo muscles

**Figure 1:**
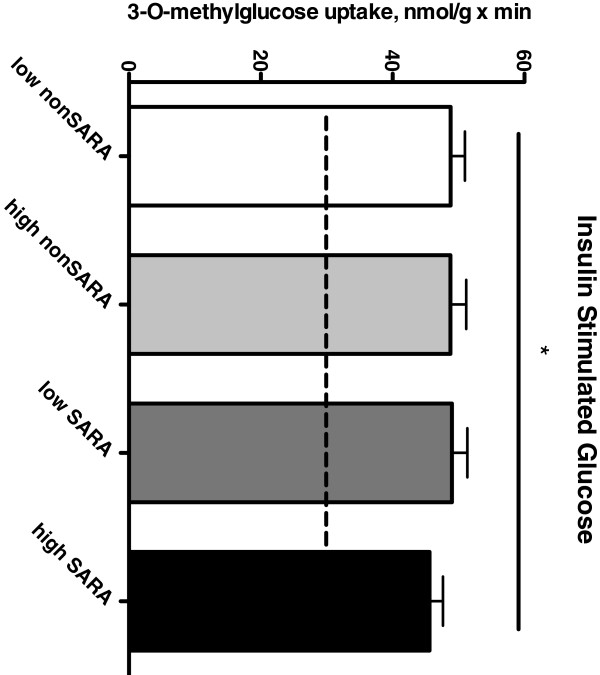
**Insulin stimulated glucose uptake in isolated soleus muscle.** Data are presented as mean plus standard error. Dashed line represents average basal (unstimulated) glucose uptake in all groups. *significantly different from basal (unstimulated) glucose uptake (p < 0.05). SOL strips were equilibrated for 30 minutes in gassed KHB (0.1% fatty acid free BSA) at 30C containing 8 mM glucose and 32 mM mannitol, in the absence or presence of insulin (10 mU/mL). Strips were washed twice (10 min each) with glucose-free KHB (4 mM pyruvate, 36 mM mannitol). After washing, SOL strips were incubated for 20 min (insulin-stimulated, 10 mU/mL) or 40 min (basal) in KHB (4 mM pyruvate, 8 mM 3-O-[^3^H] methyl-D-glucose (800 μCi/mmol), 28 mM [^14^C]mannitol (60 μCi/mmol)].

**Figure 2:**
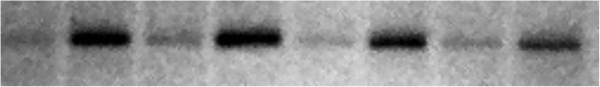
**Representative blot for Akt serine phosphorylation in incubated soleus muscle under basal and insulin stimulated conditions**. Order of Lanes: low control butter (basal), low control butter (insulin), high control butter (basal), high control butter (insulin), low SARA butter (basal), low SARA butter (insulin), high SARA butter (basal), high SARA butter (insulin).

Basal glucose transport in isolated SOL muscle did not differ amongst dietary groups (Figure 1). Insulin-stimulated glucose transport was significantly increased above basal rates (p < 0.05), but was not different across treatments. Phosphorylation (activation) of the serine residue on Akt, a key upstream protein of the insulin signalling pathway, was also increased significantly with insulin treatment (p < 0.05), but there were no differences amongst dietary treatments (Table 5; Figure 2).

**Table 5:** Akt serine phosphorylation in incubated soleus muscle under basal and insulin stimulated conditions

	**Basal condition**	**Insulin-stimulated condition**
Low control butter diet	100 ± 10 (13)	220 ± 19^c^ (13)
High control butter diet	100 ± 16 (15)	260 ± 29^c^ (15)
Low SARA butter diet	97 ± 14 (15)	254 ± 31^c^ (15)
High SARA butter diet	120 ± 19 (14)	252 ± 26^c^ (14)

Data are expressed in arbitary densiometry units relative to the low control butter diet (set to 100). Data are presented as mean plus/minus standard error. Sample size is shown in parentheses. ^c^significantly different from basal condition (p < 0.05).

### Longer term effects of SARA vs. non-SARA butter on whole body glucose, insulin and pyruvate tolerance tests

#### Rodent feed intake and body mass

During the 8 weeks of high-fat feeding, the growth curves were similar between groups and there was no statistical difference between the group means of final body weight (~340 g). The average energy intake over the 8 week period, when normalized to body weight, was nearly identical in all groups (~0.3 kilocalories/g BW/day, or ~102 kcal in total per day).

#### Fasting blood glucose

Similar to that reported with the shorter intervention, there were no significant differences in fasting blood glucose concentrations between groups following 8 weeks of their respective diets (LFD, 5.22 ± 0.19 mM; control butter, 5.30 ± 0.11 mM; SARA butter, 5.54 ± 0.16 mM; LARD, 5.48 ± 0.27 mM).

#### Tolerance tests and glucose area under the curve (AUC)

**Figure 3:**
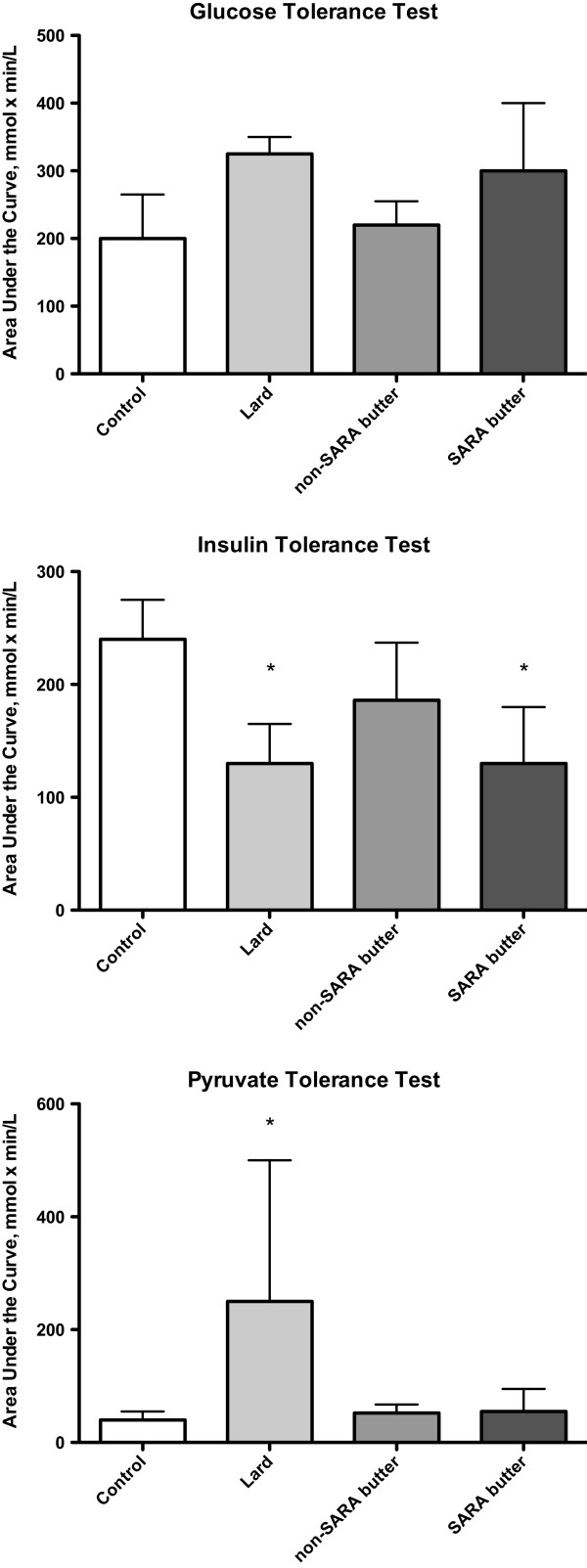
**Calculated area under the curve for glucose, insulin and pyruvate tolerance tests.** Data are presented as mean plus standard error. *significantly different from control (low fat butter) group (p < 0.05). *Glucose tolerance test.* Rats were fasted for 6 hours. A glucose bolus (2 g glucose/kg body weight) was administered by intraperitoneal injection and blood glucose concentrations were evaluated at 15, 30, 45, 60, 90 and 120 minutes post injection. *Insulin tolerance test.* Rats were fasted for 3 hours. Blood glucose levels were determined at 10, 20, 30, 45, 60, 90 and 120 minutes after an intraperitoneal insulin injection (0.75 U insulin/kg body weight). *Pyruvate tolerance test.* Rats were fasted for 6 hours. A pyruvate bolus (2 g pyruvate/kg body weight, adjusted to pH 7.35) was administered by intraperitoneal injection and blood glucose levels were evaluated at 15, 30, 45, 60, 90 and 120 minutes post injection.

Total area under the curve (AUC) for the glucose tolerance tests did not show any significant differences between dietary groups (Figure 3). Response to the insulin and pyruvate tolerance tests, however, did differ between groups. Total glucose AUC during the insulin tolerance tests was significantly reduced in both the LARD and SARA groups compared to the LFD group (p < 0.05), indicating a lower rate of glucose clearance i.e. reduced insulin sensitivity. The AUC of the glucose response during the pyruvate tolerance test was significantly greater in the LARD group compared to LFD (p < 0.05), but the butter groups were not affected.

## Discussion

This study was conducted to evaluate the potential negative health effects relating to insulin sensitivity of naturally occurring dietary CLA_t10,c12_, given the studies showing that supplemental doses of this isomer have a negative impact on insulin resistance in humans [3-6,18]. To our knowledge, this is the first study to examine the metabolic consequences of consuming naturally altered CLA_t10,c12_ content in a food source. To that end, we produced a 60% high fat diet with the fat component composed almost entirely of butter produced from milk collected from dairy cows suffering from SARA, a condition that can sometimes result from the practice in the dairy industry of feeding a high grain, low forage diet to milk-producing cows [12-14]. This butter represents, in reality, what is probably the most naturally enriched source of CLA _t10,c12_ available for human consumption. In this study, we examined the impact of shorter (4 weeks) and longer (8 weeks) term consumption of a diet highly enriched in CLA _t10,c12_ in lean healthy rats. Specifically, we examined the effects of i) 4 weeks consumption of low (10% of total kcal) and high amounts (60% of total kcal) of control (non-SARA) and SARA butter based diets on plasma glucose and insulin, as well as insulin signaling and glucose uptake in isolated rodent skeletal muscle, and ii) 8 weeks consumption of high amounts of control and SARA butter (60% of total kcal) on whole body glucose, insulin and pyruvate tolerance. The second intervention was longer in duration (8 vs. 4 weeks) in order to increase the opportunity to observe any potential detrimental effect of the CLA _t10,c12_ -enriched diet if such an effect existed.

Results from the present study indicate that within a relatively short period of time (4 weeks), there was a significant main effect for treatment (SARA vs. non-SARA) on blood glucose and plasma insulin (p < 0.05). That is, blood glucose and plasma insulin were greater in the SARA vs. non-SARA conditions, regardless of the amount of fat/butter in the diet. However, this effect was small in magnitude. Furthermore, based on the individual groups, there were no significant differences in fasting glucose or insulin concentrations. This was also confirmed in the second, longer term (8 week) intervention following which no significant change in fasting blood glucose was detected. Furthermore, the ex vivo stimulation of muscle glucose uptake and phosphorylation of Akt in response to a maximal insulin concentration was not affected by consuming butter for 4 weeks with a greater content of CLA_t10,c12_. Finally, tolerance tests (glucose, insulin and pyruvate) were performed after consuming 8 weeks of the low fat, and various high fat diets. Calculated AUC demonstrated significant impairment, or worsening, of insulin and pyruvate tolerance tests in the LARD fed animals, as would be predicted. SARA butter fed animals also demonstrated an impaired insulin tolerance test relative to the low fat fed group, but glucose and pyruvate tolerance were not adversely affected.

Collectively, our results suggest that in contrast to the findings of studies feeding higher amount of purified CLA_t10,c12_, the consumption of a diet containing naturally elevated amounts of CLA_t10,c12_ poses relatively little risk in terms of impaired muscle insulin response and whole body glucose tolerance. This may be due to several reasons including the relatively lower consumption of CLA in the current study (0.18 to 0.55 g CLA/100 g diet) compared to previous rodent studies using supplemental CLA (1.5 g/100 g diet). Furthermore, the relative abundance of all CLA isomers increased in the SARA butter such that the ratio between CLA_c9,t11_ and CLA_t10,c12_ was approximately 11:1, which is considerably greater than the 1:1 ratio of supplemented CLA. In addition, the duration of CLA consumption may also be a factor. Other studies have reported effects of CLA_t10,c12_ in mice, at similar concentrations to ours (0.5%) over a longer time course (6 months), including increases in muscle mass [19] and decreases in whole body insulin response [20]. In the present study, one of the limitations was the availability of our custom manufactured butter, which necessitated a somewhat shorter time course of feeding. Therefore, we cannot be certain as to whether a longer period of feeding CLA might not have demonstrated a clearer negative outcome.

High dairy intake has been associated with a reduced risk for type 2 diabetes [21,22] without increasing the risk of cardiovascular disease [23]. A recent study demonstrated that high consumption of low fat dairy lowered fasting insulin by 9% and insulin resistance by 11% in obese individuals [24]. An obvious limitation of our current study is that the sole source of dietary fat was butter. Few studies focus on butter as the sole source of dietary fat. One study demonstrated that healthy men consuming a high fat, butter-based diet supplemented with 5.5 g/day of mixed isomer CLA showed elevated markers of lipid peroxidation compared to butter alone, but no significant differences in fasting insulin, glucose or insulin resistance; however, there was no non-butter control used in this study [25]. In rats, the addition of milk to a diet high in sucrose was able to improve insulin sensitivity [26]. Dairy fat is complex in composition and contains numerous bioactive components that may potentially impact insulin sensitivity. For example, butter contains the short chain fatty acid butyric acid, which when supplemented in the diets of obese, high fat-fed mice improves fasting glucose, insulin and insulin tolerance [27]. In the current study, we specifically attempted to increase the content of the CLA _t10,c12_ isomer relative to that of the CLA_c9,t11_ isomer. However, it is possible that changes in other components of butter may have influenced the outcome. Indeed, the lack of any clear negative metabolic consequence to increased CLA_t10,c12_ may be due in part to compensatory effects due to altered amounts of other components, including other CLA isomers which were all observed to increase in the SARA butter. While this may make interpretation more difficult in terms of cause and effect, it does point to the need to study the effects of altered CLA content in the context of natural foods, and not merely isolated CLA isomer supplements.

Finally, it should be acknowledged that the choice of model will likely have significant bearing on the outcome. The majority of rodent studies examining the impact of CLA supplementation have utilized lean mice and obese fa/fa Zucker rats. In the current study, we did not use mice as they demonstrate a rather unique lipoatrophic response to CLA supplementation [7-9]. It is also mice which consistently show impairments in insulin sensitivity with CLA supplementation [7,8,20]. Although we plan to examine the impact of feeding our altered butters on fa/fa Zucker rats, we decided to first examine the effect of naturally altered CLA on lean rats, as they are a less “extreme” model. Less information is available regarding the effects of CLA supplementation on lean rats. However, in the Zucker rat, a commonly used rodent model of obesity and insulin resistance, CLA supplementation is generally observed to improve insulin sensitivity and inflammation [28,29]. Therefore, it is possible the general lack of negative consquences to feeding high amount of SARA butter in the current study is due to the choice of lean rats as a model i.e. that mice are more susceptible to the effects of CLA_t10,c12_ than are lean rats. Nonetheless, rats have been used by many laboratories including our own to study the effects of high fat diets on insulin sensitivity and in our opinion, represent a valid choice of species.

## Conclusion

In conclusion, the outcome of this study does not indicate an increased risk of glucose intolerance, or skeletal muscle insulin resistance in healthy rodents consuming a natural dietary fat with a higher than normal CLA_t10,c12_ content. This finding, combined with the fact that much smaller amounts of butter would be consumed in humans, as well as the relatively modest effects on insulin resistance of pharmaceutical doses in human clinical trials [3-6,18], suggests that the elevated CLA_t10,c12_ content of dairy products are unlikely to pose a risk to healthy individuals. Nonetheless, the potentially adverse effects of CLA_t10,c12_ may be more pronounced in those already overweight or obese, compared to lean individuals [5,18,30,31]. Therefore it may be prudent to evaluate the effects of high CLA_t10,c12_ butter in a model of established obesity, or in high risk groups such as those with existing insulin resistance or type 2 diabetes.

## Abbreviations

CLA: Conjugated linoleic acid; SARA: Subacute rumen acidosis; SOL: Soleus; LFDS: Low fat diet; GTT: Glucose tolerance test; ITT: Insulin tolerance test; PTT: Pyruvate tolerance test; SFA: Saturate fatty acid; PUFA: Polyunsaturated fatty acid; MUFA: Monounsaturated fatty acid; AUC: Area under the curve.

## Competing interests

The authors declare that they have no competing interests.

## Authors’ contributions

AS conducted rodent feeding experiments, analyzed data, performed statistical analyses and helped to draft the manuscript. LH assisted with cow feeding and milk collection, performed rodent feeding experiments, analyzed data, performed statistical analyses and helped to draft the manuscript. OA performed feeding of cows, milk collection and fatty acid analyses. IR helped perform rodent experiments determining glucose uptake and insulin signaling. TM assisted with oral tolerance tests and helped to interpret this data. DCW, BM and DD oversaw all aspects of the experiments, helped to interpret data and drafted the manuscript. All authors read and approved the final manuscript.
